# Clinical characteristic of prodromal symptoms between bipolar I and II disorder among Chinese patients: a retrospective study

**DOI:** 10.1186/s12888-021-03295-y

**Published:** 2021-05-31

**Authors:** Qian Zhao, Tong Guo, Yang Li, Lei Zhang, Nan Lyu, Amanda Wilson, Xuequan Zhu, Xiaohong Li

**Affiliations:** 1 The National Clinical Research Center for Mental Disorders & Beijing Key Laboratory of Mental Disorders, Beijing Anding Hospital, Capital Medical University, Beijing, China; 2 Beijing Advanced Innovation Center for Big Data Based Precision Medicine, Beihang University, Beijing, China; 3 Division of Psychology, Faculty of Health and Life Sciences, De Montfort University, Leicester, UK

**Keywords:** Bipolar II disorder, Bipolar I disorder, Prodromal symptoms, Early recognition, Depression, Mania

## Abstract

**Background:**

This study aimed to identify the clinical characteristic of prodromal symptoms in Chinese patients with bipolar disorder (BD), prior to the first affective episode. It further aimed to characterize the prodromal traits between bipolar disorder type I (BD-I) and type II (BD-II).

**Methods:**

120 individuals with BD-I (*n* = 92) and BD- II (*n* = 28) were recruited to the study. Semi-structured interviews were then administered to evaluate prodromal symptoms in patients, within 3 years of BD onset, by using the Bipolar Prodrome Symptom Scale-Retrospective (BPSS-R).

**Results:**

In the prodromal phase of the first depressive episode, patients with BD-II experienced more prodromal symptoms (*p* = 0.0028) compared to BD-I. Additionally, more frequent predictors were reported in patients with BD-II than BD-I including educational and occupational dysfunction (*p* = 0.0023), social isolation (*p* < 0.001), difficulty making decisions (*p* = 0.0012), oppositionality (*p* = 0.012), and suspiciousness/persecutory ideas (*p* = 0.017). There were also differences in the duration of the precursors. The duration of “weight loss or decrease in appetite” (*p* = 0.016) lasted longer in patients with BD-I, while “obsessions and compulsions” (*p* = 0.023) started earlier in patients with BD-II and occurred during the pre-depressive period. The prevalence and duration of each reported prodrome, preceding a first (hypo) manic episode, showed no difference between patients with BD-I and BD-II.

**Conclusions:**

Specific affective, general, or psychotic symptoms occurred prior to both affective episodes. The characteristic of prodromal symptoms were key predictors for later episodes of BD including attenuated mania-like symptoms, subthreshold depressed mood, mood swings/lability, and anxiety. In the pre-depressive state, when compared to BD-II, BD-I presented with more prodromal symptoms in nonspecific dimensions, which indicated the substantial burden of BD-II. In conclusion, this study extends the understanding of the characteristics of prodromes of BD-I and BD-II.

**Supplementary Information:**

The online version contains supplementary material available at 10.1186/s12888-021-03295-y.

## Background

Bipolar disorder (BD) is a common, chronic, and highly morbid disorder characterized by affective lability, which often runs a relapsing and remitting course, affecting 2–3% of the general population worldwide [1, 2]. The diversity of BD lies in the many forms of the disorder, including, depressive and manic or hypomanic episodes, mixed emotional states, rapid cycling, and psychosis between episodes. Patients vary greatly in the severity of their symptoms, the duration of episodes, the number of episodes, the degree of recovery between episodes, and the pattern of polarity [3, 4]. Due to the complexity of the clinical presentation of BD, there is a significant delay in diagnosis. Therefore, early recognition and intervention are important to improve the prognosis, provide intervention, and decreases the burden of the disorder.

Current evidence suggests that BD has a progressive nature [5–7], therefore supporting the theory that milder phases of the condition gradually progress to the full onset of the disorder [8]. The milder phases include the emergence of prodromal symptoms, which are symptoms and signs prior to episode onset; these symptoms are considered an ideal intervention state, as patients in early stages are more likely to be responsive to treatment and therefore may need less complex interventions [9, 10]. For this reason, the identification of risk factors, or prodromal symptoms, are important and have received increasing attention from researchers. In particular, disruptive behavior disorders, major depressive episodes, and subthreshold manic episodes were all associated with the development of BD among at-risk youth in the Pittsburgh Bipolar Offspring Study (BIOS) [11]. Anxiety/depression, affective lability, and subsyndromal manic symptoms were further established to be the strongest predictors of BD in the study [12]. In a longitudinal study with 107 Dutch adolescent who were bipolar offspring, again subthreshold manic experiences were confirmed to be the strongest predictor of BD conversion and depressive symptoms were considered to be a significant predictor for the onset of the first mood related episode [13].

Risk factors contribute to identify individuals at high risk of conversion to BD. Having a family history of BD is one of the widely recognized risk factors. Previous studies have established that the children of parents who have early-onset BD probands, also have an increased risk of BD in adulthood [12, 14]. Furthermore, research has identified that an earlier age of onset for depression, the presence of psychotic symptoms, and functional impairment robustly predicting factors of conversion [15, 16]. Environmental factors, such as life events [17], lifetime sexual abuse [18] and substance misuse [19], also play a role in defining the risk stage implicated in prodromal signs.

Most studies of the BD prodromes have used unstructured questionnaires or chart reviews and have not evaluated onset patterns systematically. The Bipolar Prodrome Symptom Scale-Retrospective (BPSS-R), is a promising screening tool to assess behavioral and symptomatic changes using semi-structured interviews [20]. Studies that have completed a detailed evaluation using the BPSS-R show that mood swings were a precursor, evolving into subthreshold (hypo) manic symptoms of subsequent BD [21]. A further study also revealed that the development of symptoms, prior to a first manic episode, were gradual in the majority of patients (88.5%), with the mean duration of progression being 10.3 ± 14.4 months. Additionally, more than half of the youth reported to have subthreshold manic symptoms, decreased school/work functioning, mood swings, and depressed mood [22]. By using a combination of the temperament scales and BPSS-R scale [23], cyclothymic temperament was found to be a significant factor to identify people at-risk of developing BD-I and -II, which may contribute to an increase in the predictive validity of prodromal symptoms.

BD-I and BD- II are the most common subtypes of BD. They vary considerably, with differences in symptomatology, management, and prognosis. Some studies revealed that BD-II when compared to BD-I presented with more severe depressive episodes, more comorbidity with anxiety disorders, and a greater risk of suicide attempts [24]. Several features associated with BD-I include greater atypical symptoms, prevalence of psychotic symptoms during a depressive episodes [25], and a greater number of annual shifts in symptom polarity [26]. These differences between BD-I and-II may begin very early in the disorder and even present as predictors for different types of BD, which are differences that require further exploration.

In consideration of the importance of prodromal symptoms for early intervention and variability between BD-I and BD-II, the researchers conducted a retrospective study to identify prodromal symptoms in patients with BD prior to the first full depressive and the first hypo/manic episode. The study was conducted with Chinese patients and further characterized the prodromal traits, in detail, between BD-I and BD-II patients.

## Material and methods

### Study design

This retrospective study enrolled 120 Chinese patients with BD, including 92 with BD-I and 28 with BD-II. Participants were recruited from Beijing Anding Hospital, a University affiliated teaching hospital in China. All participants completed the BPSS-R. The researchers explored clinical characteristic of prodromal symptoms in patients with bipolar disorder (BD), prior to the first affective episode, and further compared the prodromal traits between bipolar disorder type I (BD-I) and type II (BD-II).

### Study sample

All participants completed the clinical interview, and met the inclusion criteria, which was: 1) diagnosis of BD; 2) in clinical remission from BD; 3) the first (hypo) manic episode was within three years of the research study. The clinical status and evaluations of participants were validated by two of the senior attending psychiatrists from the hospital. The Chinese Structured Clinical Interview for Diagnostic and Statistical Manual (DSM)-IV-TR axis 1 disorders (SCID) was used to confirm a BD diagnosis [27]. A standardized questionnaire was designed to collect demographic data. The Hamilton Depression Scale-17 (HAMD-17) [28, 29] and the Young Mania Rating Scale (YMRS) [30] were used to rate symptom severity. The current remission status of participants was confirmed by the HAMD-17, including those with a score less than 17, and the YMRS, including those with a score less than 12. The BPSS-R interview was conducted by a trained psychiatrist with all participants to both trace and assess prodromal symptoms prior to a first depressive or (hypo) maniac episode. As the study included patients with BD-II as well, the prodromal symptoms prior to a first manic and hypomanic episode were merged for BD-II patients. Those who had drug or alcohol dependence or substance use issues within the last year, had a history of stroke, a head injury, had epilepsy, and/or had a presence of progressive systemic disorders were excluded from participation.

### Instruments

BPSS-R is a semi-structured interview instrument, which was designed based on the severity of downward criteria in the DSM-IV, it is based on available diagnostic and psychopathology assessment scales for BD and major depressive disorder (MDD). Consent was obtained to use the scale from Correll [20], the author of the original scale. In order to avoid the potential impact of cultural differences, the researchers used a back-translation method. The scale was translated into Chinese by one translator and then translated back into English again by an independent translator who was blind to the original scale. The back-translation version had the same meaning as the original, which ensured the adaption was culturally relevant and in a comprehensible form for Chinese psychiatrists and patients, while at the same time replicable of the original scale.

The BPSS-R assesses the onset pattern, duration, severity, and frequency of 39 symptoms and signs that emerge or become worse prior to the first (hypo) manic or major depressive episode. Prodromal symptom severity is divided into 3 levels (1 = mild, 2 = moderate, and 3 = severe). Symptom frequency is divided into 4 levels (1 = rarely, 2 = relapse/intermittent, 3 = continuous, and 4 = lifetime/ personality). The pre-onset symptoms are categorized into four subgroups: mania index (extremely energetic/active; overly cheerful, happy, on top of the world; racing thoughts; overly talkative; decreased need for sleep; irritability or anger; overly self-confident; trouble concentrating; physically agitated; increased sexual energy; reckless or dangerous behavior; risky sexual behavior); depression index (depressed mood; tiredness or lack of energy; reduction of enjoyment/ interest; trouble concentrating; insomnia; feeling of worthlessness or guilt; weight loss or decrease in appetite; slow movement; suicidality; physical agitation; sleeping too much; attempting suicide; weight gain or increasing in appetite); general index (educational and occupational dysfunction; anxiety or nervousness; social isolation; frequent mood swings/lability; difficulty making decisions; obsessions and compulsions; losing temper frequently or trouble controlling anger; day/night sleep reversal; self-injurious behavior; oppositionality; giddy, clownish; increased creativity); and psychosis index (suspiciousness/persecutory ideas; strange or unusual ideas; difficulty thinking or communicating clearly; hallucinations).

### Data analysis

All data were analyzed using SAS 9.4 for Windows. The Shapiro-Wilk Test was used to check normality in data distribution. Continuous variables were analyzed by an Independent Sample t-Test for normal distribution. Continuous variables that lacked normal distribution and presented with variance and non-homogeneity were analyzed using the Wilcoxon (Mann-Whitney U) Rank Sum Test. Continuous data were described using mean and standard deviation. Categorical data was indicated by number and percentage (%) and were then compared by the Chi-square Test or Fisher Exact Probability Test between the two groups. A two-tailed *p*-value was utilized for various statistical analyses, and *p* < 0.05 was considered to be statistically significant.

## Results

### Study population

Demographic and clinical characteristics of the 120 patients with BD are summarized in Table 1. 76.7% of patients were diagnosed with BD-I, 23.3% with BD-II. 65% of participants were male and the mean age was 26.5 years old (SD = 10.0). Average years of schooling were 12.3 years (SD = 3.3) and more than half (62.5%) of the participants were unmarried. The mean age at first episode was 26.3 years old (SD = 10.6) in BD-I and 23.1 years old (SD = 8.7) in BD-II patients. The mean age at the first episode was 25.6 (SD = 10.2) and the average age to seek medical aid was 26.2 (SD = 10.2). In addition, the substantial lag between onset of the disorder and clinical intervention was more than half a year (7.2 ± 12.6 months).

**Table 1:** Demographic Characteristics and Disorder Characteristics of BD Patients

Variables	Total (*n* = 120)	BD-I (*n* = 92)	BD-II(*n* = 28)	t /χ2	*P*-value
Age (years, mean ± SD)	26.5 ± 10.0	27.4 ± 10.4	24.7 ± 8.4	1.26	0.21
Male sex (n, %)	78(65.0%)	61(66.3%)	17(60.7%)	0.29	0.59
Education (years, mean ± SD)	12.3 ± 3.3	12.4 ± 3.3	12.3 ± 3.3	0.15	0.89
Marital status (n, %)					
Unmarried	75(62.5%)	54(58.7%)	21(75.0%)	2.43	0.12
Married/ Divorced/other	45(37.5%)	38(41.3%)	7(25.0%)		
Age at first episode (years, mean ± SD)	25.6 ± 10.2	26.3 ± 10.6	23.1 ± 8.7	1.47	0.14
Age at first clinical visit (years, mean ± SD)	26.2 ± 10.2	26.8 ± 10.6	23.9 ± 8.4	1.59	0.17
Lag between first episode and clinical visit (months, mean ± SD)	7.2 ± 12.6	6.8 ± 10.0	10.8 ± 16.7	0.20	0.65
Disorder course (months, mean ± SD)	20.3 ± 15.7	19.4 ± 16.3	23.1 ± 13.7	2.90*	0.089
Remission (n, %)					
Partial remission	68(56.7%)	53(58.2%)	15(53.6%)	0.1907	0.66
Full remission	52(43.3%)	39(42.4%)	13(46.4%)		
vTotal scores of HAMD-17 (mean ± SD)	1.4 ± 1.9	1.3 ± 1.8	1.8 ± 2.2	0.90*	0.34
Total scores of YMRS (mean ± SD)	0.7 ± 1.1	0.7 ± 1.2	0.4 ± 1.4	3.8472	0.05

Abbreviations: BD, Bipolar Disorder; SD, Standard Deviation; HAMD-17, The Hamilton Depression Scale17; YMRS, Young Mania Rating Scale

The disorder cause, defined as the period from the date of first BD-I and BD-II diagnosis to the time of the interview was 20.3 months (SD = 15.7). The lag in presentation and disorder onset was longer in patients with BD-II than BD-I (10.8 ± 16.7 months vs. 6.8 ± 10.0 months; 23.1 ± 13.7 months vs. 19.4 ± 16.3 months), although it was not statistical significance. 58.2% of patients with BD-I and 53.6% of patients with BD-II achieved partial remission. The average of total scores of HAMD-17 and YMRS were low, 1.4 (SD = 1.9) and 0.7 (SD = 1.1) respectively, which was in accordance with the enrollment criteria (current absence of major manic or depressive symptoms). Participants who achieved full remission were 42.9% in BD-I and 41.8% in BD-II groups. Overall, demographic characters and clinical variables showed no differences between BD-I and BD-II patients.

In patients with BD-I, there were 67.4% (62/92) and 94.6% (87/92) of patients reporting prodromal symptoms prior to the first depressive episode and the first manic episode respectively. In patients with BD-II, 78.6% (22/28) and 92.2% (26/28) of patients reported prodromal symptoms prior to the first depressive episode and the first hypomanic episode. Most symptoms in the prodromal state were reported to persist through the first full-brown affective episode and specific symptom items are shown in Supplementary Table 1 and Supplementary Table 2. All patients who reported at least one symptom belonging to the subgroup domains were considered in each index’s total score.

### Prodromal symptoms prior to the fist depressive episode

#### Prevalence

The prevalence of prodromal symptoms between patients with BD-I and BD-II prior to first depressive episode are displayed in Table 2. 54.4% patients with BD-I and 85.7% of patients with BD-II reported early manifestations prior to a first full depressive episode, and more BD-II patients experienced prodromal symptoms in this study (*p* = 0.0028). Although the proportion of the presence of each item in the depressive index showed no difference between BD-I and BD-II groups, the depressive index total was detected to have a significant difference between BD-I and BD-II groups (*p* = 0.0063). Most patients (53.3% for BD-I and 82.1% for BD-II) reported depression-related symptoms in the prodromal phase of the first depressive episode. The five most frequent prodromal symptoms in the depressive index in BD patients were “depressed mood” (BD-I: 40.2%, BD-II: 46.4%), “reduction of enjoyment/ interest” (30.4% for BD-I, 32.1% for BD-II), “tiredness or lack of energy” (BD-I: 26.1%, BD-II: 35.7%), “trouble concentrating” (BD-I: 23.9%, BD-II: 42.9%), and “insomnia” (BD-I: 21.7%, BD-II: 39.3%).

**Table 2:** Reported Prevalence and Duration of Prodromal Symptoms between Patients with BD-I and BD-II Prior to First Depressive Episode

Prodromal Symptom characteristics	Prevalence (Yes)					Duration (Weeks)				
	BD-I(*n* = 92)		BD-II(*n* = 28)		*P* value	BD-I(*n* = 92)		BD-II(*n* = 28)		*P* value
	N	%	N	%		Mean	SD	Mean	SD	
**Mania Symptom Index**										
Extremely energetic/active	3	3.3	1	3.6	1.00 a	8.6	0.0	0.0	0.0	n.a.
Overly cheerful, happy, on top of the world	3	3.3	0	0.0	1.00a	10.1	5.0	0.0	0.0	n.a.
Racing thoughts	2	2.2	0	0.0	1.00 a	32.4	27.5	25.9	0.0	1.00
Overly talkative	3	3.3	3	10.7	0.14 a	13.0	0.0	25.9	24.4	1.00
Decreased need for sleep	2	2.2	2	7.1	0.23 a	0.0	0.0	0.0	0.0	n.a.
Irritability or anger	11	12.0	6	21.4	0.22	11.7	9.1	5.4	3.1	0.14
Overly self-confident	1	1.1	1	3.6	0.41	17.3	0.0	43.2	0.0	0.19
Physical agitated	2	2.2	0	0.0	1.00a	10.8	10.3	4.3	0.0	0.062
Increased sexual energy	0	0.0	1	3.6	0.23 a	4.3	0.0	43.2	0.0	n.a.
Reckless or dangerous behaviors	0	0.0	0	0.0	n.a.	0.0	0.0	0.0	0.0	n.a.
Risky sexual behavior	0	0.0	0	0.0	n.a.	0.0	0.0	0.0	0.0	n.a.
Trouble concentrating	22	23.9	12	42.9	0.05	13.9	12.9	11.8	14.9	0.52
**Mania Index total**	29	31.5	16	57.1	0.014*					
**Depression Symptom Index**										
Depressed mood	37	40.2	13	46.4	0.56	14.4	14.0	7.0	3.8	0.062
Tiredness or lack of energy	24	26.1	10	35.7	0.32	12.3	10.2	6.7	3.0	0.11
Reduction of enjoyment/ interest	28	30.4	9	32.1	0.90	15.4	16.7	7.9	6.6	0.21
Trouble concentrating	22	23.9	12	42.9	0.05	13.9	12.9	11.8	14.9	0.52
Insomnia	20	21.7	11	39.3	0.06	15.4	14.9	12.2	13.6	0.55
Feeling of worthlessness or guilt	11	12.0	7	25.0	0.13^a^	9.6	7.5	9.2	7.5	1.00
Weight loss or decrease in appetite	8	8.7	5	17.9	0.18	17.3	16.2	5.6	3.3	0.016*
Physically slowed down	6	6.5	5	17.9	0.13 ^a^	9.2	9.1	4.3	0.0	0.36
Thinking about suicide	6	6.5	3	10.7	0.43	8.0	8.1	10.8	3.1	0.15
Sleeping too much	8	8.7	5	17.9	0.18	6.5	4.3	17.3	23.1	0.51
Attempting suicide	3	3.3	2	7.1	0.33^a^	4.3	0.0	4.3	0.0	1.00
Weight gain or increasing in appetite	4	4.4	1	3.6	1.00 ^a^	23.3	18.0	23.8	27.5	0.85
**Depression Index Total**	49	53.3	23	82.1	0.0063**					
**General Symptom Index**										
Educational and occupational dysfunction	19	20.7	14	50.0	0.0023**	9.7	8.2	4.3	0.0	0.27
Anxiety or nervousness	24	26.1	11	39.3	0.18	13.2	12.6	11.8	13.8	0.74
Social Isolation	14	15.2	15	53.6	< 0.001***	4.3	0.0	0.0	0.0	n.a.
Frequent mood swings/lability	10	10/9	5	17.9	0.34 a	14.4	15.4	6.5	4.6	0.054
Difficulty making decisions	6	6.5	9	32.1	0.0012 **^a^	11.5	9.9	11.0	12.6	0.88
Obsessions and compulsions	5	5.4	5	17.9	0.05 ^a^	6.9	3.9	21.6	14.5	0.023*
Losing temper frequently or trouble controlling anger	9	9.8	2	7.1	1.00a	11.5	8.5	8.6	7.5	0.4962
Day/night sleep reversal	3	3.3	2	7.1	0.33a	13.7	15.3	25.9	24.1	0.3452
Self-injurious behavior (no intent to kill self)	3	3.3	2	7.1	0.33	8.6	6.1	5.8	2.5	0.5541
Oppositionality	0	0.0	3	10.7	0.012 ^a^	5.8	2.5	4.3	0.0	
Giddy, clownish	2	2.2	1	3.6	0.55 ^a^	0.0	0.0	19.4	21.4	n.a.
Increased creativity	3	3.3	1	3.6	1.00 ^a^	4.3	0.0	8.6	0.0	n.a.
**General Index Total**	39	42.4	22	78.6	0.0008***					
**Psychosis Symptom Index**										
Suspiciousness/persecutory ideas	3	3.3	5	17.9	0.017*	4.3	0.0	7.6	6.5	n.a.
Strange or unusual ideas	5	5.4	1	3.6	1.00 ^a^	30.3	30.6	23.8	27.5	
Difficulty thinking or communicating clearly	17	18.5	7	25.0	0.45	10.9	8.6	5.9	4.0	
Hallucinations	3	3.3	0	0.0	1.00^a^	0.0	0.0	0.0	0.0	n.a.
**Psychosis Index Total**	21	22.8	11	39.3	0.085					
**BPSS-R Total**	50	54.4	24	85.7	0.0028					

Abbreviations: BD, Bipolar Disorder; SD, Standard Deviation; BPSS-R, Bipolar Prodrome Symptom Scale-Retrospective

n.a. = not applicable

*P < 0.05 **P < 0.01 ***P < 0.001

a: Fisher exact test

Prior to the first depressive episode, the prevalence of mania index total differed between patients with BD-I and BD-II (*p* = 0.014). Although each item in the mania symptom index showed no statistical difference between the two BD groups, some items deserve attention. The item of “irritability or anger” and “overly talkative” tended to occurr more in BD-II group (21.4%;10.7%).

The prevalence for the general symptom index total was significantly different during the depressive prodromal phase between the two BD groups (BD-I: 42.4% vs. BD-II: 78.6%, *p* < 0.001). Four self-reported items of the general index, namely educational and occupational dysfunction (*p* = 0.0023), social isolation (*p* < 0.001), difficulty making decisions (*p* = 0.0012) and oppositionality (*p* = 0.012), were reported to occur significantly more often in BD-II patients, prior to the first depressive episode. Although, psychotic symptoms were not specific in BD, the frequency of occurrence of suspiciousness/persecutory ideas was significantly higher in BD- II patients (17.9%) than BD-I patients (3.3%) during the pre-depressed phase (*p* = 0.017).

#### Duration

The comparison of the duration of prodromal symptoms prior to the fist depressive episode between BD-I and BD- II patients is shown in Table 2. The duration of each item during the manic and psychotic index symptoms did not differ significantly between the two BD groups. The symptom of “weight loss or decrease in appetite” in the depressive symptom index started significantly earlier and before the first full depressive episode among BD-I patients compared to BD- II patients (17.3 ± 16.2 weeks vs. 5.6 ± 3.3 weeks, *p* = 0.016). The duration of “obsessions and compulsions” in the general symptom index in the pre-depressive stage was reported to last longer in BD- II patients than in BD-I patients. (21.6 ± 14.5 weeks vs. 6.9 ± 3.9 weeks, *p* = 0.023).

### Prodromal symptoms prior to the first (hypo) mania episode

#### Prevalence and duration

The results of reported prevalence and duration of prodromal symptoms between patients with BD-I and BD-II prior to the first (hypo) mania episode are shown in Table 3. 81.5% of patients with BD-I and 78.6% of patients with BD-II reported early manifestations prior to the first (hypo) manic episode. There was no significant difference between BD-I and BD-II patients in the presence of each item in the four dimensions; namely mania index, depression index, general index, and psychosis index. Reported prodromal symptoms with a prevalence of more than 25% included: “extremely energetic/active” (BD-I: 34.8%, BD-II: 39.3%), “overly cheerful, happy, on top of the word” (BD-I: 29.4%, BD-II: 28.6%), “racing thoughts” (BD-I: 26.1%, BD-II: 21.4%), “overly talkative” (BD-I: 34.8%, BD-II: 32.1%), “frequent mood swings/lability” (BD-I: 30.4%, BD-II: 21.4%), and “losing temper frequently or trouble controlling anger” (BD-I: 32.6%, BD-II: 14.3%). The duration of each prodromal symptom prior to the first (hypo) manic episode presented with no difference between BD-I and BD-II groups.

**Table 3:** Reported Prevalence and Duration of Prodromal Symptoms Between Patients with BD-I and BD-II Prior to the First Mania/ Hypomania Episode

Prodromal symptom characteristics	Prevalence (Yes/No)					Duration (Weeks)				
	BD-I(*n* = 92)		BD-II(*n* = 28)		P value	BD-I(*n* = 92)		BD-II(*n* = 28)		P value
	N	%	N	%		Mean	SD	Mean	SD	
**Mania Symptom Index**										
Extremely energetic/active	32	34.8	11	39.3	0.66	8	5.8	4.9	3.6	0.10
Overly cheerful, happy, on top of the world	27	29.4	8	28.6	0.94	13.9	21.1	8.6	8.6	0.38
Racing thoughts	24	26.1	6	21.4	0.62	8.3	6.7	4.3	3.5	0.24
Overly talkative	32	34.8	9	32.1	0.80	6.5	4	8.6	8.6	0.82
Decreased need for sleep	18	19.6	5	17.9	0.84	13.7	28.5	5.2	4.7	0.45
Irritability or anger	21	22.8	6	21.4	0.88	15.8	25.4	7.2	5.2	0.54
Overly self-confident	13	14.1	3	10.7	0.64	21.6	50.3	15.6	20.9	0.53
Physical agitated	9	9.8	3	10.7	0.89	13	9.7	6.1	4.9	0.18
Increased sexual energy	3	3.3	3	10.7	0.14	5.8	3.5	6.5	7.5	0.74
Reckless or dangerous behaviors	10	10.9	3	10.7	1.00^a^	21.6	40.4	10.1	10.9	0.90
Risky sexual behavior	3	3.3	3	10.7	0.14^a^	2.2	3.1	10.8	15.3	0.68
Trouble concentrating	14	15.2	4	14.3	1.00^a^	17.3	14	8.6	6.1	0.22
**Mania Index total**	65	70.7	18	64.3	0.52	15.8	28.6	12.2	13.8	0.83
**Depression Symptom Index**										
Depressed mood	7	7.6	4	14.3	0.28^a^	30.8	36.7	10.4	4.9	0.37
Tiredness or lack of energy	6	6.5	2	7.1	1.00^a^	15.6	12.4	8.6	4.3	0.55
Reduction of enjoyment/ interest	6	6.5	1	3.6	1.00^a^	25.2	22.1	4.3	6.1	0.18
Trouble concentrating	14	15.2	4	14.3	1.00^a^	17.3	14	8.6	6.1	0.22
Insomnia	18	19.6	3	10.7	0.40^a^	11.7	12.5	5.8	6.6	**0.35**
Feeling of worthless or guilty	5	5.4	2	7.1	0.66 ^a^	10.4	10.8	6.5	2.5	1.00
Weight loss or decrease in appetite	5	5.4	3	10.7	0.39 ^a^	17.3	21.8	8.6	5.3	0.91
Physically slowed down	3	3.3	0	0.0	1.00 ^a^	18.7	28.8	–	–	n.a.
Thinking about suicide	1	1.1	1	3.6	0.41^a^	10.1	13.9	4.3	6.1	0.77
Sleeping too much	5	5.4	1	3.6	1.00^a^	30.3	49	10.1	13.9	0.46
Attempting suicide	1	1.1	0	0.0	1.00^a^	2.2	3.1	–	–	n.a.
Weight gain or increasing in appetite	5	5.4	4	14.3	0.21	16.2	24	16.2	24	1.00
**Depression Index Total**	33	35.9	12	42.9	0.50	20.3	28.1	14.4	14.7	0.95
**General Symptom Index**										
Educational and occupational dysfunction	20	21.7	2	7.1	0.081	18.5	15.6	8.6	6.1	0.37
Anxiety or nervousness	16	17.4	5	17.9	1.00^a^	38.9	50.6	13.8	16.8	0.23
Social isolation	12	13.0	1	3.6	0.30^a^	21.2	17.9	6.9	3.9	0.078
Frequent mood swings/lability	28	30.4	6	21.4	0.35	17.7	29.9	11.7	11.4	0.86
Difficulty making decisions	4	4.4	3	10.7	0.35^a^	31.3	48.4	6.1	3.9	0.45
Obsessions and compulsions	7	7.6	2	7.1	1.00^a^	26.5	39.7	6.5	5.6	0.44
Losing temper frequently or trouble controlling anger	30	32.6	4	14.3	0.060	12.3	23.6	7.2	4.3	0.83
Day/night sleep reversal	5	5.4	1	3.6	1.00a	11.7	17.9	10.1	13.9	0.72
Self-injurious behavior (no intent to kill self)	0	0.0	0	0.0	n.a	79.9	106.9	4.3	6.1	0.44
Oppositionality	15	16.3	3	10.7	0.58^a^	22.6	31.1	5.8	6.6	0.13
Giddy, clownish	15	16.3	3	10.7	0.56^a^	10.8	14.9	7.2	6.6	0.93
Increased creativity	12	13.0	5	17.9	0.54^a^	21.1	50.5	12.1	17.7	0.82
**VGeneral Index Total**	62	67.4	17	60.7	0.51	20.1	35.6	14.5	14.4	0.70
**Psychosis Symptom Index**										
Suspiciousness/persecutory ideas	20	21.7	4	14.3	0.39	13.9	26.3	4.3	3.1	0.22
Strange or unusual ideas	21	22.8	4	14.3	0.33	20.7	30	5.8	5	0.23
Difficulty thinking or communicating clearly	9	9.8	3	10.7	1.00^a^	18	17.2	0	.	n.a.
Hallucinations	5	5.4	1	3.6	1.00a	10.1	9	2.2	3.1	0.37
**Psychosis Index Total**	32	34.8	7	25.0	0.33	16.8	24.7	5.0	3.3	0.11
**BPSS-R Total**	75	81.5	22	78.6	0.73	18.4	32.6	15.7	15.0	0.48

Abbreviations: BD, Bipolar Disorder; SD, Standard Deviation; BPSS-R, Bipolar Prodrome Symptom Scale-Retrospective

n.a. = not applicable

*P < 0.05 **P < 0.01 ***P < 0.001

a: Fisher exact test

## Discussion

To the researchers’ knowledge, this is the first applied study of the BPSS-R among Chinese patients with BD to identify prodromal symptoms prior to first full affective episode. As well as being the first study to explore the prodromal differences between BD-I and BD-II patients, which has received little attention previously. In the prodromal phase of the first depressive episode, patients with BD-II experienced more prodromal symptoms (*p* = 0.0028) compared to BD-I. In patients with BD-II more frequent predictors included educational and occupational dysfunction (*p* = 0.0023), social isolation (*p* < 0.001), difficulty making decisions (*p* = 0.0012), oppositionality (*p* = 0.012), and suspiciousness/persecutory ideas (*p* = 0.017) than those reported in BD- I. There were also differences in the duration of the precursors. The duration of “weight loss or decrease in appetite” (*p* = 0.016) lasted longer in patients with BD-I, while “obsessions and compulsions” (*p* = 0.023) started earlier in patients with BD-II in the pre-depressive period. The prevalence and duration of each reported prodrome preceding a first (hypo) mania episode showed no difference between patients with BD-I and BD-II.

In this study, for the first depressive episode, patients with BD-II had more prodromes in each subdomain than patients with BD-I. BD-II patients had more occurrences of educational and occupational dysfunction, social isolation, difficulty making decisions, oppositionality, and suspiciousness/persecutory ideas before the first full-blown depressive episode. The patients also revealed a history of subsyndromal depressive symptoms, which were predictors for BD, which is further supported by a previous study [31]. BD-II has been established to be more severe and challenging compared to BD-I in several studies, such as higher rates of early onset, longer BD duration, higher prevalence of psychiatric comorbidity, more prior mood episodes, as well as more current depressive symptoms [24, 32]. This study’s findings indicate that having a substantial subthreshold for depressive symptoms entails a greater potential of development of BD-II. Indeed, improved knowledge of prodromal symptoms for BD-II could help identify the disorder earlier and facilitate enhanced treatment for such individuals.

A history of mood swings/lability prior to the a first (hypo) manic episode was reported by more than a quarter of patients in this study, with a larger proportion in BD-I patients compared to BD-II patients (30.4% vs. 21.4%). This is consistent with previous studies that mood swings/lability can represent a precursor or part of a prodrome evolving into a full (hypo) maniac episode both in youth and adults [33, 34]. Previous studies have also found that lifetime subsyndromal hypomanic symptoms predicted later development of BD spectrum disorders [19, 35, 36]. This study confirmed that subsyndromal hypomania, such as “extremely energetic/active”, “overly cheerful, happy, on top of the world”, “racing thoughts”, and “overly talkative” was reported as pre-(hypo) manic symptoms by most of the patients. It is worth noting that 28.6% of BD-II patients in this study reported attenuated mania-like symptoms before full expression of a depressive episode, and that previous studies further supported that depression as associated with hypomanic features increased the risk of later BD [37, 38].

Anxiety symptoms, psychotic features, and other nonspecific psychopathological presentations emerged as antecedents to the onset of BD in several studies [11, 13, 39–41]. As this study presented, anxiety or nervousness was common in the prodromal stage in both BD-I and BD-II patients. In addition, in this study BD-II patients presented more general symptoms compared to BD-I patients, prior to a first depressive episode, including “educational and occupational dysfunction”, “social isolation”, “difficulty making decisions” and “oppositionality”. These nonspecific symptoms are worthy of attention in future clinical work as theymay be relatively sensitive in predicting later onset of BD-II. Psychotic features have also been identified as clinical precursors of BD and have indicated the progression of BD conversion in the clinical phases [40, 41]. In this study, it is interesting to note that BD-II patients reported more psychotic symptoms before the first depressive episode, which extends the understanding of characteristics of prodromes of BD-II. It further confirmed that there exists a substantial burden of the various dimensional symptoms in BD-II.

In this study, the prodromal symptoms persisted for a period prior to the first affective episode. Several studies confirm the progressive nature of BD. The duration of prodromes was relatively shorter in our study (2–32 weeks, with most lasting 10–15 weeks), compared with those in previous studies [22, 35, 42, 43]. A meta-analysis reported that the average duration of the prodromal period preceding an initial mood episode (manic or depressive) was 107.9 ± 91.5 months, although the average prodrome duration to an initial manic episode was 10.6 ± 10.9 months, which is obviously shorter than the average duration considering both depressive and maniac episodes [43]. In this study, the data set of participants was smaller. Therefore the researchers speculated that there were several potential relative reasons for the differences between the studies’ findings. Firstly, many patients with BD-I reported an initial manic episode in this study. Therefore, bias in sample composition may have had an impact on the results. Secondly, quite a few people in China lack knowledge about mental disorders, which limits their ability to recognize early symptoms. Thirdly, due to the reserved culture, Chinese people might be more prone to suppress their inner emotions. Fourthly, recall bias might exist in a retrospective study, which is mainly based on patients’ subjective report, even if the course of the disorder was limited to within 3 years of onset. Despite this, the duration of the prodromes could provide clinicians a chance to conduct earlier interventions. Notably, in this study “Weight loss or decrease in appetite” and “obsessions and compulsions” started earlier in BD-II patients preceding a first depressive episode. Previous studies reported that individuals with obsessive-compulsive disorder had a 13-fold increased risk of developing BD [37], and obsessive and compulsive symptoms were more common in BD- II than in BD-I [44], indicating that the presence of obsessive-compulsive disorder may be an independent risk factor for the later development of BD, especially for BD- II.

There are several limitations to our study. First, the study was based on a mixed sample of patients with BD-I and BD-II. The sample size was relatively small, which might limit the generalization of the findings. However, in a recent large-scare epidemiological study of mental disorders in China [45], the life-time prevalence of BD-I and BD-II were 0.4% (106/32552) and less than 0.1% (12/32552) respectively. The prevalence of BD-I is then considered to be 8 times more than BD-II in China, consistent with this study in which more BD-I patients than BD-II patients were enrolled. Our study also focused on the comparison of prodromal symptoms, which are relatively common in BD patients. Some specific features for each subtype of BD were detected in this study, although the small sample size was not adequately powered to detect differences with small effect sizes. Secondly, although the retrospective cross-sectional design of this study might cause recall bias; the researchers did restrict the course of the disorder within three years of onset, which could decrease the recall bias. Thirdly, family history of mental disorder(s) was not collected in this study, which could be an important predictive factor to characterize prodromal symptoms. Another limitation is that patients with borderline personality disorder may not have been picked up during the screening process. Although open questions about personality and extreme behavior were asked to patients and their family members to exclude potential individuals with personality disorders, this was a rough screening. The Structured Clinical Interview for DSM-IV Axis II Disorders should be used to identify borderline personality disorder in the future.

In addition, the researchers used standardized diagnostic tools and semi-fixed assessment tools to diagnose and evaluate patients, but failed to set up a control group, such as monophasic depression, schizophrenia, and healthy controls. Including control groups would improve rigor, as prodromal symptoms may overlap between different disorders and also occur in normal populations. Some prospective studies have assessed mood symptoms in healthy individuals. A study with college students who had “subsyndromal bipolar” were more likely to develop BD or major depressive disorder [46]. A dose-response relationship between the number of both manic and depressive symptoms and persistence was observed in adolescents and young adults [47]. In bipolar offspring in the study, an individual risk calculator was developed to predict the 5-year risk of developing BD [34]. In non-BD individuals a lower incidence of prodromal symptoms and shorter duration of symptoms was found. Overall, the prodromal symptoms had a predictive effect on BD.

## Conclusions

In conclusion, specific affective symptoms, as well as general or psychotic symptoms, occurred prior to both affective episodes in BD. Characteristics of prodromal symptoms included attenuated mania-like symptoms, subthreshold depressed mood, mood swings/lability, and anxiety were key predictors for later onset of BD. In non-specific dimensions, the pre-depressive state presented more prodromal symptoms in BD-II compared to BD-I, indicating the substantial disorder burden of BD-II. Overall, this study extended the understanding of characteristics of prodromes of BD-I and BD-II.

## Supplementary Information

**Additional file 1.**

## Data Availability

The datasets generated and analyzed during the current study are not publicly available due to ethical restrictions and personal data protection, but are available from the corresponding author Dr. Xiaohong Li on reasonable request.

## Abbreviations

BD: Bipolar disorder
BPSS-R: The Bipolar Prodrome Symptom Scale-Retrospective
SCID: Structured Clinical Interview for Diagnostic and Statistical Manual (DSM)-IV-TR axis 1 disorders
HAMD-17: The Hamilton depression scale-17
YMRS: The Young Mania Rating Scale
SD: Standard deviation
