# Supplementary material for: Clinical characteristic of prodromal symptoms between bipolar I and II disorder among Chinese patients: a retrospective study
Source: BMC Psychiatry. 2021 May 31;21:275. doi: 10.1186/s12888-021-03295-y (PMC8168043; doi:10.1186/s12888-021-03295-y)
Supplement: Supplementary file 1 — Additional file 1. [file 12888_2021_3295_MOESM1_ESM.docx]

Supplementary table 1. The prevalence of prodromal symptoms preceding the first depressive episode.

|  | **BD-I（n=92）** | | | | **BD-II（n=28）** | | | |
| --- | --- | --- | --- | --- | --- | --- | --- | --- |
| Prodromal symptom characteristics | Reported prodrome | | Prodrome preceding the first depressive episode | | Reported prodrome | | Prodrome preceding the first depressive episode | |
|  | N | % | N | % | N | % | N | % |
| **Mania Symptom Index** |  |  |  |  |  |  |  |  |
| Extremely energetic/active | 3 | 3.3 | 2 | 2.2 | 1 | 3.6 | 1 | 3.6 |
| Overly cheerful, happy, on top of the world | 3 | 3.3 | 1 | 1.1 | 0 | 0.0 | 0 | 0.0 |
| Racing thoughts | 2 | 2.2 | 2 | 2.2 | 0 | 0.0 | 0 | 0.0 |
| Overly talkative | 3 | 3.3 | 1 | 1.1 | 3 | 10.7 | 0 | 0.0 |
| Decreased need for sleep | 2 | 2.2 | 2 | 2.2 | 2 | 7.1 | 2 | 7.1 |
| Irritability or anger | 11 | 12.0 | 8 | 8.7 | 6 | 21.4 | 4 | 14.3 |
| Overly self-confident | 1 | 1.1 | 0 | 0.0 | 1 | 3.6 | 0 | 0.0 |
| Physical agitated | 2 | 2.2 | 2 | 2.2 | 0 | 0.0 | 0 | 0.0 |
| Increased sexual energy | 0 | 0.0 | 0 | 0.0 | 1 | 3.6 | 0 | 0.0 |
| Reckless or dangerous behaviors | 0 | 0.0 | 0 | 0.0 | 0 | 0.0 | 0 | 0.0 |
| Risky sexual behavior | 0 | 0.0 | 0 | 0.0 | 0 | 0.0 | 0 | 0.0 |
| Trouble concentrating | 22 | 23.9 | 15 | 16.3 | 12 | 42.9 | 10 | 35.7 |
| **Mania Index total** | 29 | 31.5 |  |  | 16 | 57.1 |  |  |
| **Depression Symptom Index** |  |  |  |  |  |  |  |  |
| Depressed mood | 37 | 40.2 | 26 | 28.3 | 13 | 46.4 | 12 | 42.9 |
| Tiredness or lack of energy | 24 | 26.1 | 19 | 20.7 | 10 | 35.7 | 9 | 32.1 |
| Reduction of enjoyment/ interest | 28 | 30.4 | 20 | 21.7 | 9 | 32.1 | 7 | 25.0 |
| Trouble concentrating | 22 | 23.9 | 15 | 16.3 | 12 | 42.9 | 10 | 35.7 |
| Insomnia | 20 | 21.7 | 12 | 13.0 | 11 | 39.3 | 9 | 32.1 |
| Feeling of worthless or guilty | 11 | 12.0 | 9 | 9.8 | 7 | 25.0 | 7 | 25.0 |
| Weight loss or decreasing in appetite | 8 | 8.7 | 7 | 7.6 | 5 | 17.9 | 4 | 14.3 |
| Physically slowed down | 6 | 6.5 | 5 | 5.4 | 5 | 17.9 | 4 | 14.3 |
| Thinking about suicide | 6 | 6.5 | 4 | 4.3 | 3 | 10.7 | 3 | 10.7 |
| Sleeping too much | 8 | 8.7 | 5 | 5.4 | 5 | 17.9 | 4 | 14.3 |
| Attempting suicide | 3 | 3.3 | 2 | 2.2 | 2 | 7.1 | 2 | 7.1 |
| Weight gain or increasing in appetite | 4 | 4.4 | 2 | 2.2 | 1 | 3.6 | 1 | 3.6 |
| **Depression Index Total** | 49 | 53.3 |  |  | 23 | 82.1 |  |  |
| **General Symptom Index** |  |  |  |  |  |  |  |  |
| Educational and occupational dysfunction | 19 | 20.7 | 12 | 13.0 | 14 | 50.0 | 10 | 35.7 |
| Anxiety or nervousness | 24 | 26.1 | 14 | 15.2 | 11 | 39.3 | 9 | 32.1 |
| Social Isolation | 14 | 15.2 | 9 | 9.8 | 15 | 53.6 | 13 | 46.4 |
| Frequent mood swings/lability | 10 | 10.9 | 7 | 7.6 | 5 | 17.9 | 4 | 14.3 |
| Difficulty making decisions | 6 | 6.5 | 3 | 3.3 | 9 | 32.1 | 8 | 28.6 |
| Obsessions and compulsions | 5 | 5.4 | 4 | 4.3 | 5 | 17.9 | 3 | 10.7 |
| Losing temper a lot or trouble  controlling anger | 9 | 9.8 | 6 | 6.5 | 2 | 7.1 | 2 | 7.1 |
| Day night sleep reversal | 3 | 3.3 | 2 | 2.2 | 2 | 7.1 | 1 | 3.6 |
| Self-injurious behavior (no intent to kill self) | 3 | 3.3 | 2 | 2.2 | 2 | 7.1 | 2 | 7.1 |
| Oppositionality | 0 | 0.0 | 0 | 0.0 | 3 | 10.7 | 2 | 7.1 |
| Giddy, clownish | 2 | 2.2 | 1 | 1.1 | 1 | 3.6 | 1 | 3.6 |
| Increased creativity | 3 | 3.3 | 1 | 1.1 | 1 | 3.6 | 0 | 0.0 |
| **General Index Total** | 39 | 42.4 |  |  | 22 | 78.6 |  |  |
| **Psychosis Symptom Index** |  |  |  |  |  |  |  |  |
| Suspiciousness/persecutory ideas | 3 | 3.3 | 3 | 3.3 | 5 | 17.9 | 2 | 7.1 |
| Strange or unusual ideas | 5 | 5.4 | 4 | 4.3 | 1 | 3.6 | 1 | 3.6 |
| Difficulty thinking or communicating clearly | 17 | 18.5 | 11 | 12.0 | 7 | 25.0 | 6 | 21.4 |
| Hallucinations | 3 | 3.3 | 3 | 3.3 | 0 | 0.0 | 0 | 0.0 |
| **Psychosis Index Total** | 21 | 22.8 |  |  | 11 | 39.3 |  |  |
| **BPSS-R Total** | 50 | 54.4 |  |  | 24 | 85.7 |  |  |

Supplementary table 2. The prevalence of prodromal symptoms preceding the first (hypo)manic episode.

|  | **BD-I（n=92）** | | | | **BD-II（n=28）** | | | |
| --- | --- | --- | --- | --- | --- | --- | --- | --- |
| Prodromal symptom characteristics | Reported prodrome | | Prodrome preceding the first manic episode | | Reported prodrome | | Prodrome preceding the first hypomanic episode | |
|  | N | % | N | % | N | % | N | % |
| **Mania Symptom Index** |  |  |  |  |  |  |  |  |
| Extremely energetic/active | 32 | 34.8 | 18 | 19.6 | 11 | 39.3 | 6 | 21.4 |
| Overly cheerful, happy, on top of the world | 27 | 29.4 | 16 | 17.4 | 8 | 28.6 | 3 | 10.7 |
| Racing thoughts | 24 | 26.1 | 14 | 15.2 | 6 | 21.4 | 4 | 14.3 |
| Overly talkative | 32 | 34.8 | 22 | 23.9 | 9 | 32.1 | 5 | 17.9 |
| Decreased need for sleep | 18 | 19.6 | 11 | 12.0 | 5 | 17.9 | 4 | 14.3 |
| Irritability or anger | 21 | 22.8 | 17 | 18.5 | 6 | 21.4 | 4 | 14.3 |
| Overly self-confident | 13 | 14.1 | 9 | 9.8 | 3 | 10.7 | 2 | 7.1 |
| Physical agitated | 9 | 9.8 | 7 | 7.6 | 3 | 10.7 | 2 | 7.1 |
| Increased sexual energy | 3 | 3.3 | 1 | 1.1 | 3 | 10.7 | 3 | 10.7 |
| Reckless or dangerous behaviors | 10 | 10.9 | 7 | 7.6 | 3 | 10.7 | 1 | 3.6 |
| Risky sexual behavior | 3 | 3.3 | 3 | 3.3 | 3 | 10.7 | 2 | 7.1 |
| Trouble concentrating | 14 | 15.2 | 9 | 9.8 | 4 | 14.3 | 4 | 14.3 |
| **Mania Index total** | 65 | 70.7 |  |  | 18 | 64.3 |  |  |
| **Depression Symptom Index** |  |  |  |  |  |  |  |  |
| Depressed mood | 7 | 7.6 | 3 | 3.3 | 4 | 14.3 | 2 | 7.1 |
| Tiredness or lack of energy | 6 | 6.5 | 3 | 3.3 | 2 | 7.1 | 1 | 3.6 |
| Reduction of enjoyment/ interest | 6 | 6.5 | 4 | 4.3 | 1 | 3.6 | 1 | 3.6 |
| Trouble concentrating | 14 | 15.2 | 9 | 9.8 | 4 | 14.3 | 4 | 14.3 |
| Insomnia | 18 | 19.6 | 11 | 12.0 | 3 | 10.7 | 3 | 10.7 |
| Feeling of worthless or guilty | 5 | 5.4 | 3 | 3.3 | 2 | 7.1 | 2 | 7.1 |
| Weight loss or decreasing in appetite | 5 | 5.4 | 4 | 4.3 | 3 | 10.7 | 1 | 3.6 |
| Physically slowed down | 3 | 3.3 | 2 | 2.2 | 0 | 0.0 | 0 | 0.0 |
| Thinking about suicide | 1 | 1.1 | 1 | 1.1 | 1 | 3.6 | 0 | 0.0 |
| Sleeping too much | 5 | 5.4 | 3 | 3.3 | 1 | 3.6 | 1 | 3.6 |
| Attempting suicide | 1 | 1.1 | 1 | 1.1 | 0 | 0.0 | 0 | 0.0 |
| Weight gain or increasing in appetite | 5 | 5.4 | 3 | 3.3 | 4 | 14.3 | 4 | 14.3 |
| **Depression Index Total** | 33 | 35.9 |  |  | 12 | 42.9 |  |  |
| **General Symptom Index** |  |  |  |  |  |  |  |  |
| Educational and occupational dysfunction | 20 | 21.7 | 15 | 16.3 | 2 | 7.1 | 2 | 7.1 |
| Anxiety or nervousness | 16 | 17.4 | 8 | 8.7 | 5 | 17.9 | 5 | 17.9 |
| Social Isolation | 12 | 13.0 | 8 | 8.7 | 1 | 3.6 | 1 | 3.6 |
| Frequent mood swings/lability | 28 | 30.4 | 18 | 19.6 | 6 | 21.4 | 4 | 14.3 |
| Difficulty making decisions | 4 | 4.4 | 4 | 4.3 | 3 | 10.7 | 3 | 10.7 |
| Obsessions and compulsions | 7 | 7.6 | 4 | 4.3 | 2 | 7.1 | 1 | 3.6 |
| Losing temper frequently or trouble controlling anger | 30 | 32.6 | 24 | 26.1 | 4 | 14.3 | 2 | 7.1 |
| Day night sleep reversal | 5 | 5.4 | 2 | 2.2 | 1 | 3.6 | 0 | 0.0 |
| Self-injurious behavior (no intent to kill self) | 0 | 0.0 | 0 | 0.0 | 0 | 0.0 | 0 | 0.0 |
| Oppositionality | 15 | 16.3 | 13 | 14.1 | 3 | 10.7 | 3 | 10.7 |
| Giddy, clownish | 15 | 16.3 | 9 | 9.8 | 3 | 10.7 | 2 | 7.1 |
| Increased creativity | 12 | 13.0 | 5 | 5.4 | 5 | 17.9 | 3 | 10.7 |
| **General Index Total** | 62 | 67.4 |  |  | 17 | 60.7 |  |  |
| **Psychosis Symptom Index** |  |  |  |  |  |  |  |  |
| Suspiciousness/persecutory ideas | 20 | 21.7 | 13 | 14.1 | 4 | 14.3 | 4 | 14.3 |
| Strange or unusual ideas | 21 | 22.8 | 16 | 17.4 | 4 | 14.3 | 3 | 10.7 |
| Difficulty thinking or communicating clearly | 9 | 9.8 | 5 | 5.4 | 3 | 10.7 | 3 | 10.7 |
| Hallucinations | 5 | 5.4 | 3 | 3.3 | 1 | 3.6 | 1 | 3.6 |
| **Psychosis Index Total** | 32 | 34.8 |  |  | 7 | 25.0 |  |  |
| **BPSS-R Total** | 75 | 81.5 |  |  | 22 | 78.6 |  |  |
